# Experimental investigation of flat fan nozzle V-cut depths and its impact on spray characteristics

**DOI:** 10.1038/s41598-025-05319-z

**Published:** 2025-07-02

**Authors:** Waqas Mughal, Ji Pei, Junping Liu, Wenjie Wang, Jalab Hussain, Imran Ahmed Samo, Xie Rongjun, Yongqiang Zhang

**Affiliations:** 1 Research Center of Fluid Machinery Engineering and Technology, Jiangsu University, Zhenjiang, 212013 China; 2 Department of Mechanical Engineering, Quaid-e-Awam University of Engineering, Science and Technology, Nawabshah, Sindh Pakistan; 3 Department of Environmental Engineering, Quaid-e-Awam University of Engineering, Science and Technology, Nawabshah, Sindh Pakistan

**Keywords:** V-Cut depth, Spray angle, V-Cut angle, Spray volume distribution, Droplet size, High speed imaging, Engineering, Mechanical engineering

## Abstract

In this study, five locally produced different V-cut flat fan nozzles named A2, B2, C2, D2 and E2 were introduced for experimental investigation on their effect on spray characteristics. The results were collected about droplet size, spray volume distribution, spray angle, sheet length, velocity, Weber number and discharge coefficient experimentally at pressures of 100 kPa, 200 kPa, 300 kPa and 400 kPa respectively. To evaluate nozzle performance under varying pressures, each parameter had its own experimental setup. The results showed that V-cut depth greatly impacts flat fan nozzle performance. Spray angle and droplet size were both enhanced with increasing V-cut depth. The deeper cuts improved the flow dynamics, which in turn permitted the nozzle to spray at a wider angle and with finer droplets. This study reveals that as increasing the spray angle significantly improved the spray volume distribution respectively. This allows for more uniform and wide coverage. Conversely, reducing the depth of the V-cut increases the stream velocity resulted the droplets become coarser due to insufficient shear stress. When the fluid’s velocity increases too rapidly, the shear stress does not have adequate time to effectively atomize the fluid into fine droplets. While higher velocity generally enhances shear, excessive flow speed can hinder the fluid from experiencing the appropriate shear forces at the optimal moment. As a result, instead of producing smaller droplets, the fluid ends up generating larger, coarser droplets. A mathematical model was developed from the experimental data to estimate droplet size and spray angle. The model’s predictions closely match the experimental results, demonstrating the model’s accuracy. The investigation found that the optimum V-shaped cutting depths were C2, D2, and E2, as these nozzles obtained better spray angles and showed a greatly improvement droplet size and spray volume distribution.

## Introduction

The use of pesticides is an important part of protecting crops from disease and pests which supports to reduce losses and increase productivity. There are many technological advances in integrated pest control that facilitate plant growth and development^1^. Agricultural spraying relies heavily on sprayer nozzles. Flat fan nozzles have a variety of applications, including pesticide, fertilizer, and nutrient application, surface cleaning, spray coating, and greenhouse cooling. The main objectives while design of Spray nozzle is to achieve a specific goal such as achieving homogenous distribution, consistent droplet size and precise targeting of the spray to specified locations or pests. When using insecticides with a sprayer, the atomizing properties of the nozzle are important. This has a significant impact on the effectiveness of insecticide applications. Factors such as droplet size, spray pattern and spray pressure must be taken into account. These properties directly affect the coverage and distribution of pesticides on the target surface^2,3^. The performance of a pressure-self-controlled water-pesticide integrated sprinkler was examined using an experimental approach for trellis crops^4^. Microsprinkler nozzles can meet irrigation and pesticide needs under low-pressure and medium-pressure settings, respectively^5^. The Spray characteristics parameters is a necessary parameters for evaluating the hydraulic performance of a nozzle^6^. Droplet characteristics mainly include droplet size, droplet velocity, and droplet kinetic energy^7^. Although a small spray drop shows effective depositions and coverage, but it is sensitive to drift^8^. Understanding the importance of different designs and controlling factors that affect sprinkler efficiency and uniformity are critical^9^. Droplet speed affects pesticide and nutrient application. Spray droplets with very low velocity cannot reach the desired target, meanwhile droplets with higher velocity tend to bounce back^10,11^. Therefore, it is important to understand and improve the atomization characteristics in order to effectively use pesticide sprayers in agriculture and forestry. The main purpose of this design is to achieve several objectives, such as consistent application, drift reduction, and precisely targeting the spray to a specified location or insect. Different nozzle types and sizes result in a variety of spray patterns and droplet sizes^12,13^. These factors affect the extent of plant spray coverage and increases or decreases the probability of drift. Since the 1930s, researchers have examined how nozzle shapes—round, square, rectangular, and oval—affect atomization and the advancement of liquid dispersion from two-dimensional to three-dimensional spraying studied, The correlation between pressure and droplet size was that as the spray pressure increased, the droplet size decreased. Flat fan nozzle is commonly known for its mountainous-shaped spray pattern and even distribution used for agricultural spraying and several other industrial applications^14^. Nuyttens, David, et al. conducted in-depth research on droplet characteristics using different nozzles and applied pressure. The findings underscore the significant influence of both nozzle type and size on droplet size and velocity spectra. When comparing nozzle diameters and spray pressures, cone nozzles exhibited a fine range of droplet sizes comparatively with regular flat fan nozzles, low-drift flat fan nozzles, and air injection nozzles following in that order^15^. The air-induction nozzle, which injects air into a droplet, has demonstrated efficacy in agricultural engineering. This nozzle generates larger droplets than normal flat-fan nozzles at identical spray pressure and pesticide flow rate, hence diminishing the likelihood of drift^16^. Abhishek et al. examined how nozzle type and operating pressure affected spray characteristics and discharge rate in which reduced the impact on the environment and improve the effectiveness of pesticide application for greenhouse farming. Taking into account the data and other variables, the flat fan nozzle type outperformed the hollow cone nozzle and flower nozzle (8-hole) in terms of transmittance percentages, suggesting improved coverage potential^17^.

Akbar and Abolfazl made an effort to investigate and introduce a factor that has not been considered so far – the outlet design of a flat-fan nozzle. As the outlet design of a flat-fan nozzle increases (i.e., orifice size varies depending on outlet type), the size of spray droplets decreases, causing a set-off between orifice size and outlet type that may spark new interest in the perspective of drone application^18^. Gabriela et al. assessed how spray droplet size (1) and spray nozzle type (2) influence the quantity and quality of spray deposition on peanut plants at the vegetative and reproductive growth stages. Two greenhouse studies were conducted on V1 and R1 growth stages peanut plants. Three droplet sizes—medium (fat fan XR 11002), very coarse (TT 11002), and extremely coarse (AIXR 11002)—were used in Experiment 1 treatments. Five spray nozzles were hollow cone (TXA 8002), twin fat fan (TTJ60 11002), air induction fat fan (AIXR 110025), drift guard twin fat fan (DGTJ60 11002), and extended range fat fan (XR 11003). The flat fan XR 11,003 and hollow cone nozzle TXA 8002 VK exhibited optimal spray deposition on peanut plants during the V1 and R1 growth phases^19^. Altimira et al. investigated the effect of nozzle size on flow dynamics using numerical simulations^20^. The basic characteristics of initial spraying include droplet size, droplet speed, liquid density and fan angle^21^. The V-shaped nozzle of the flat fan greatly affects the spray pattern and droplet size. This design feature includes a V-shaped groove or slot on the nozzle tip to ensure a more uniform and controlled spray. V-cuts increase application efficiency and reduce waste. However, the actual impact of droplet size also depends on by additional factors such as liquid pressure, flow rate and nozzle design. Therefore, careful optimization is important to achieve the best results under different spray conditions. This study focuses on investigating the effect of the V-shaped cutting depth of a flat fan nozzle. This is an important design feature that affects the spray’s characteristics. Variation in this parameter affects important spray performance parameters such as droplet size, spray volume distribution, spray angle, and spray sheet length. Five V-cut depth parameter were analyzed to understand how they affect the spray characteristics. Three different working pressures are used for each V-cut depth, providing a comprehensive understanding of the interaction between V-cut geometry and fluid dynamics. The experimental results regarding droplet size and spray angle are of great importance and has been used to develop efficient mathematical models to predict the droplet size and spray angle. The model provides a predictive framework for estimating drop size and spray angle based on changes in V-cut depth and operating pressure. The ability to predict spray drop size under various conditions is crucial. Optimizing the nozzle design is crucial to enhance its efficiency in the agricultural industry and other spray applications. This study provides specific information on the relationship between pressure and V-cutting depth and droplet size to measure Spray performance through developed model which meet requirements. These design parameters are a systematic approach to the design of nozzles that reduce waste and increase process efficiency.

## Materials and methods

### Theoretical calculation

**Fig. 1:**
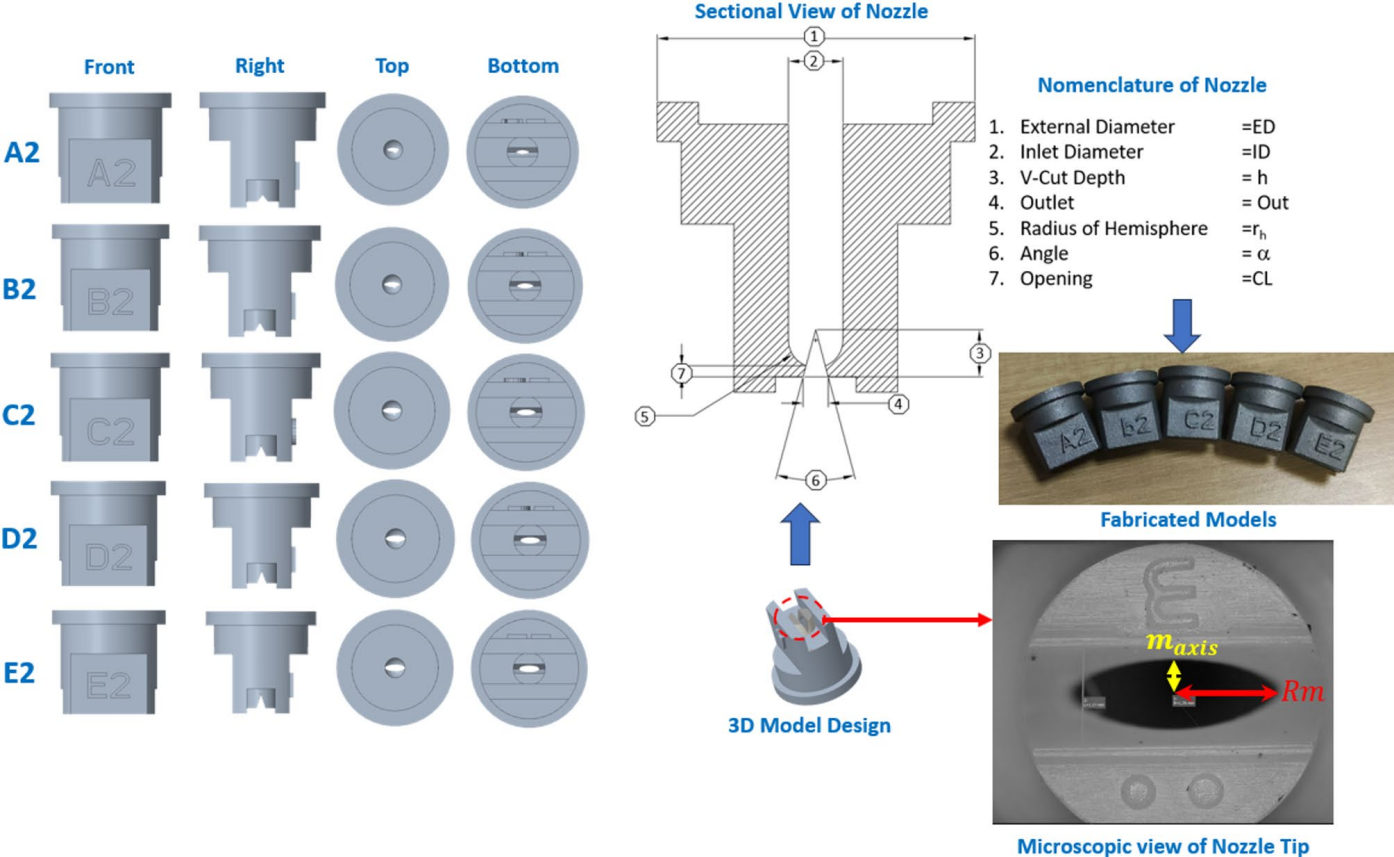
Nomenclature of produced different V-cut depth of flat fan nozzle.

This study investigates the effects of V-Cut depth and pressure on droplet size produced by a flat fan nozzle. When a V-Cut is introduced in the flat fan nozzle, the resulting cross-section of the formed spray becomes elliptical. The major and minor axes need to be determined to measure the elliptical cross-sectional area. Additionally, the lower section of the flat fan nozzle is hemispherical, as shown in Fig. 1, where dimensional parameters are given in Table 1.

As we know, the depth of the V-Cut will influence the major and minor axes of the elliptical cross-section. The major axis of this section is calculated as^22^

1 $${H_v}={r_h} - \sqrt {{r_h}^{2} - R{m^2}}$$

Where ${H_v}$ is V-Cut depth in the hemispherical section, rh is the radius of the hemisphere and Rm is radius of V-Cut or radius of major axis.

As we know that, in Flat Fan nozzle, the hemispherical section started after little above the injector surface. However, ${H_v}$ is calculated as

2 $${H_v}=h - CL$$

3 $$\backslash Rm=\sqrt {2rh{H_v}+{H_v}^{2}}$$

Similarly, for minor axis calculation

4 $${m_{axis}}=Rm\sin \left( {\frac{\alpha }{2}} \right)$$

Where, $\alpha$ is the central angle of the V-Cut. Once the major and minor axes have been evaluated, the following equation has been used to evaluate the ellipse area.

5 $$A{V_{ellipse~}}=~\pi \times Rm \times Rm~\sin \left( {\frac{\alpha }{2}} \right)$$

Simplifying

6 $$A{V_{ellipse~}}=~\pi R{m^2}\sin \left( {\frac{\alpha }{2}} \right)$$

**Table 1:** Dimensional parameters of variable V-cut developed nozzle.

Sr.	Model	Pressure kPa	Inlet diameter (ID) mm	Outlet width (out) mm	Depth of V-Cut (h) mm	Angle (α)	Opening (CL) mm
1	A2	100 ~ 400	2	1.17	0.75	76	0.40
2	B2	100 ~ 400	2	1.17	1	61	0.40
3	C2	100 ~ 400	2	1.17	1.25	50	0.40
4	D2	100 ~ 400	2	1.17	1.5	43	0.40
5	E2	100 ~ 400	2	1.17	1.75	37	0.40

The discharge coefficient (Cd) for a flat fan nozzle is an important parameter that quantifies the efficiency of a nozzle in delivering fluid from a pressurized system to the surrounding environment.

Cd is affected by factors such as nozzle geometry, edge sharpness, flow conditions (laminar or turbulent), and the nature of the fluid being released. The nozzles with sharp edges or smooth transitions exhibit a lower coefficient of discharge, whereas smooth surfaces and edges may lead to higher Cd values. The coefficient of discharge can be measured as^23^

7 $$Cd=~\frac{{{Q_{actal}}}}{{{Q_{Theoritical}}}}$$

8 $${Q_{Theoritical}}=~A{V_{ellipse~}}\sqrt {\frac{{2\Delta P}}{\rho }}$$

Whereas $A{V_{ellipse~}}$ is the area of the injector, $\rho$ density of fluid $\Delta p$ nozzle pressure difference. The nozzle discharge coefficient expresses the ratio of actual discharge value to the theory value as given in Eq. (7). The developed film velocity can be calculated as given in Eq. (9)^24^.

9 $$U=~Cd\sqrt {\frac{{2\Delta p}}{\rho }}$$

The Weber number (We) is an important dimensionless number in fluid dynamics, especially when analyzing spray nozzles. It quantifies the relative importance of inertial forces to surface tension in a fluid. This is important in understanding the atomization process, which forms small droplets from the continuous fluid flow stream. It can be measured as^25^

10 $$We=~\frac{{\rho {U^2}L}}{\sigma }$$

Whereas U is the velocity of the fluid, L is the characteristic length (typically the hydraulic diameter or some representative length scale of the hole), and $\sigma$ is the surface tension of the fluid. The outlet section of flat fan nozzle is considered as elliptical however L is characteristic length or hydraulic diameter. In order to convert L into a hydraulic diameter of the elliptical section, the following equation has been used.

11 $$L=Dh=~\frac{{4A{V_{ellipse~}}}}{p}$$

### Experimental setup

This study has investigated the effect of V-cut depth on flat fan nozzles, with an emphasis on how different V-cut depths and pressures affect spray droplet size, spray angle, sheet length, and spray coverage. Various methodologies were employed to experimentally examine these parameters, which are described in detail below.

#### Spray droplet size measurement methodology

**Fig. 2:**
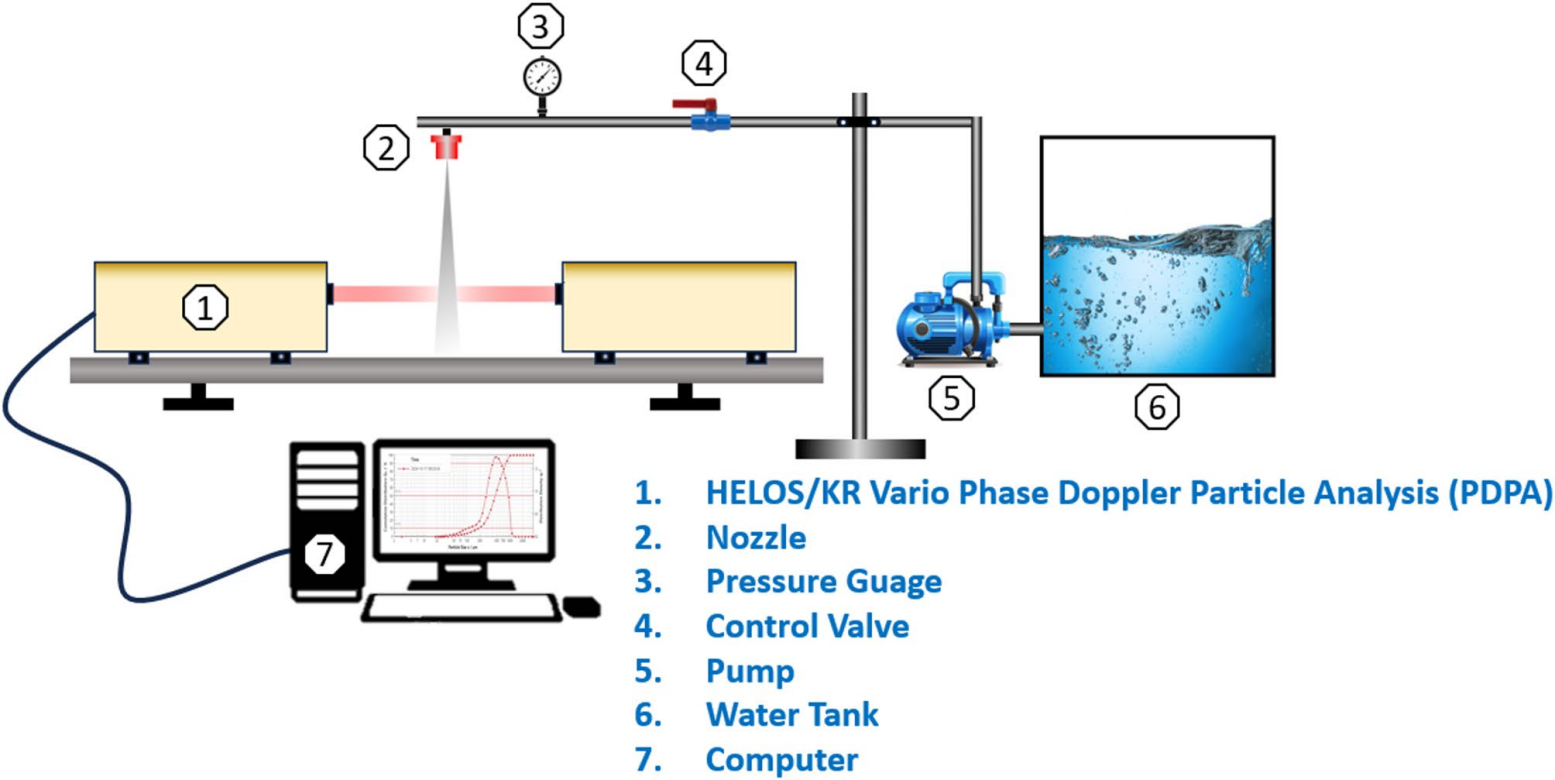
Schematic diagram of experimental setup for measurement of spray droplet size and distribution.

In this study, the spray dynamics were experimentally investigated at five different V-Cut depths with on pressures of 100, 200 and 300 kPa, respectively. The experiment was performed at the Irrigation Laboratory of the Research Center of Fluid Machinery Engineering and Technology (Jiangsu University, Zhenjiang City, Jiangsu Province, P.R China) to investigate Droplet characteristics such as DV0.1, DV0.5, and DV0.9. These droplets were analyzed using the HELOS/KR Vario Phase Doppler Particle Analysis (PDPA) instrument as shown in Fig. 2. The droplet size was measured 12 inches from the nozzle, as at this distance the sheet length produced from flat fan nozzle is fully converted into droplets. It has been clearly seen through experiments where the droplet forms through physical observation that 12 inches is the effective distance to measure the droplet size of the developed spray from the produced nozzle. In another study, the author used the SympaTec Helos laser beam instrument to measure the droplet size. The distance of 20 cm (8 inches) from the nozzle to the laser beam was used to measure the droplet size^26^. Each test was taking 10 s to measure the droplet size. Each test was conducted three times, and the findings were averaged to provide accurate results. The pressure gauge used in this study ranged from 0 to 0.6 MPa with an accuracy of 0.4%. The nozzle spray was performed using a 12 VDC double-headed diaphragm water pump, with an open flow rate of 10–12 LPM and a maximum ampere consumption of 4–7 AMP. All these experiments were conducted on a laboratory scale.

This study utilized the experimentally obtained data to develop a mathematical model to predict the value of droplet size as a function of V-cut depth and pressure.

#### Spray coverage volume distribution measurement methodology

**Fig. 3:**
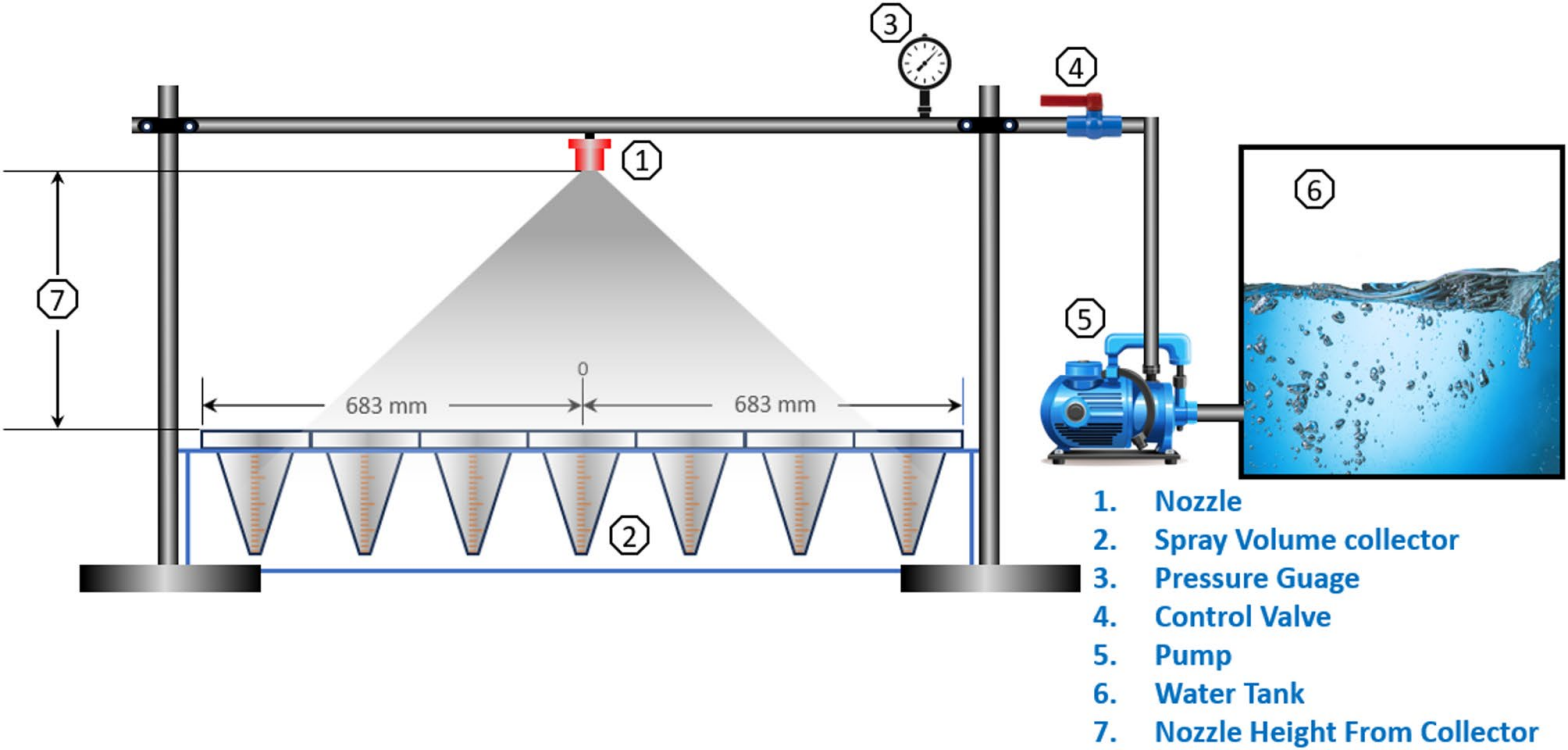
Schematic diagram of experimental setup for measurement of spray volume distribution.

Spray distribution and coverage from the pesticide flat fan nozzle are key factors in effective pest control and minimizing chemical waste. The flat fan spray head creates an even spray pattern that distributes the pesticide in a fan-like distribution, allowing for large area coverage. Spray pattern width and droplet size may vary depending on nozzle type, pressure and application speed, however in order to achieve maximum efficiency, adjusting the nozzle angle, pressure and height above the target is essential to achieve the desired delivery and reduce drift potential. This study additionally examined into the nozzle’s spray coverage, focusing on the effects of pressure and V-cut depth. At three distinct heights, H12, or 12 inches; H24, or 24 inches; and H36, or 36 inches, the spray coverage generated by the nozzle was investigated. The heights were measured from the nozzle tip to the collecting bowl, as illustrated in Fig. 3. This study utilized seven bowl collectors placed exactly below the nozzle for the measurement of spray volume and droplet collection. The distance from the nozzle’s centre to both the left and right edges was 683 mm. Generally, the spray width of a flat fan nozzle ranges from 1.1 m to 1.5 m, approximately 1500 mm. In this study, measured from the centre, both the left and right sides are 683 mm, totalling about 1400 mm, which is considered a greater coverage distance. The research examined the collection of spray volume at five distinct V-cut depths, over three pressure settings and three height changes.

#### Spray angle and sheet length measurement methodology

**Fig. 4:**
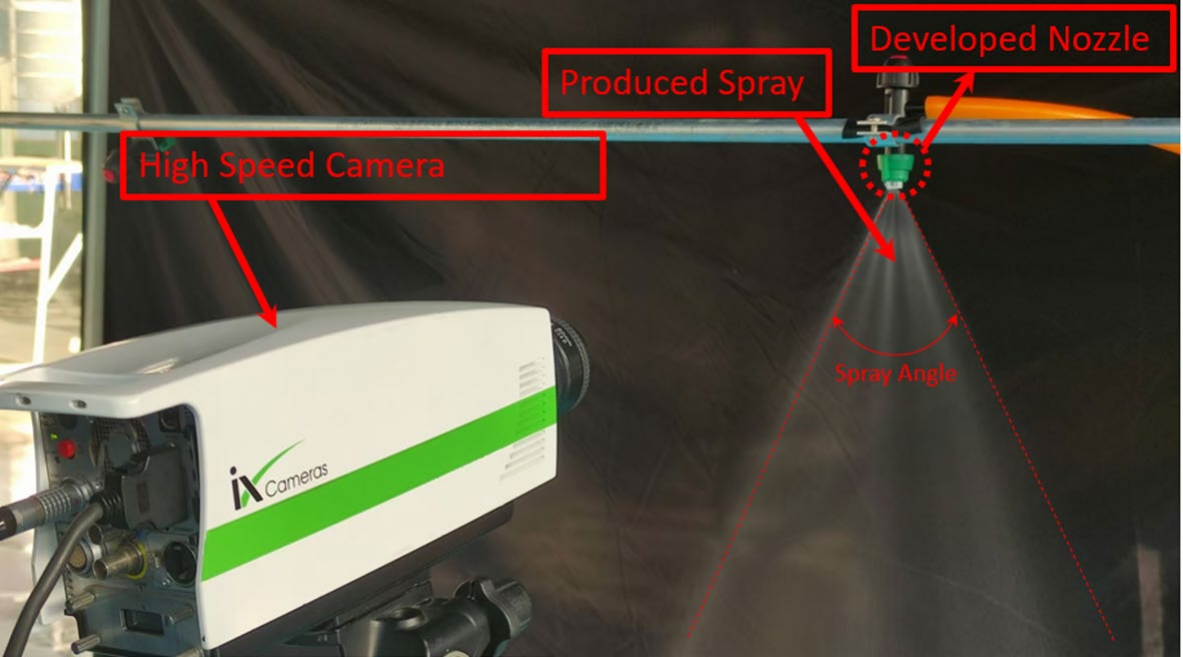
Pictorial view of experimental setup for measurement of spray angle, sheet length and spray formation using high speed camera.

The spray angle of a flat fan nozzle is important to ensure that the application of liquids such as pesticides, fertilizers or water effectively on targeted areas. A broader spray angle covers greater area, rendering it suitable for general applications or broad surfaces, whilst a smaller angle offers a more focused spray, advantageous for precision operations or targeted treatments. Sheet length breakup and droplet generation are crucial to understanding how spray nozzles, especially flat fan nozzles, distribute liquid efficiently. These mechanisms affect droplet size, spray pattern, coverage, and pesticide, fertilizer, or other fluid application efficiency. This study examined spray angle and sheet length. This investigation showed that V-Cut depth and pressure affect spray angle and sheet length. Figure 4 shows the i-SPEED 3 high-speed camera measuring spray angle and sheet length. However, high speed Videos were taken at various pressures for each V-cut depth model. The high-speed camera in this investigation is capable of recording 150,000 frames per second. The footage used in this study were collected at 10,000 FPS to illustrate sheet length breakup and Spray angle experimental measurement.

The high-speed video was initially used to acquire appropriate images in order to measure the spray angle and sheet length. Subsequently, the images were imported into AutoCAD software and scaling into the appropriate size for measuring Spray angle and Sheet length. Measurements were taken of the Spray angle and sheet length after the image had been scaled. In this study, the different V-Cut depth model is tested at different pressures to measure spray angles, sheet lengths, and other spray characteristics.

## Results and discussion

Flat fan nozzles are an essential component in agricultural irrigation systems and various fluid applications. These nozzles are designed to provide a consistent and controlled spray pattern, making them suitable for a variety of applications. These nozzles produced a wider and consistent fan-like spray pattern, which provides an even liquid distribution. This spray pattern is famous for the application of insecticides, herbicides, and fertilizers in a wide area. This consistency is important to ensure precision in application, reduce waste, and avoid under- or over the target. This can affect efficiency and environmental impact. Moreover, flat fan nozzles are very popular as they are suitable for various spray volumes and pressures. When used appropriate nozzle design parameters, the produced large droplets unaffected by air can reduce drift and increase the amount of liquid reaching the target. This study focuses on the effects of V-Cut depth and pressure on various spray parameters such as droplet size, comprehensive spray volume distribution, spray angle, sheet length, Weber number, velocity, and discharge coefficient. Below, we discuss these sub-parameters and demonstrate how they impact the spray characteristics’ performance and efficiency.

### Effect of V-cut depth and pressure on droplet size and distribution

**Fig. 5:**
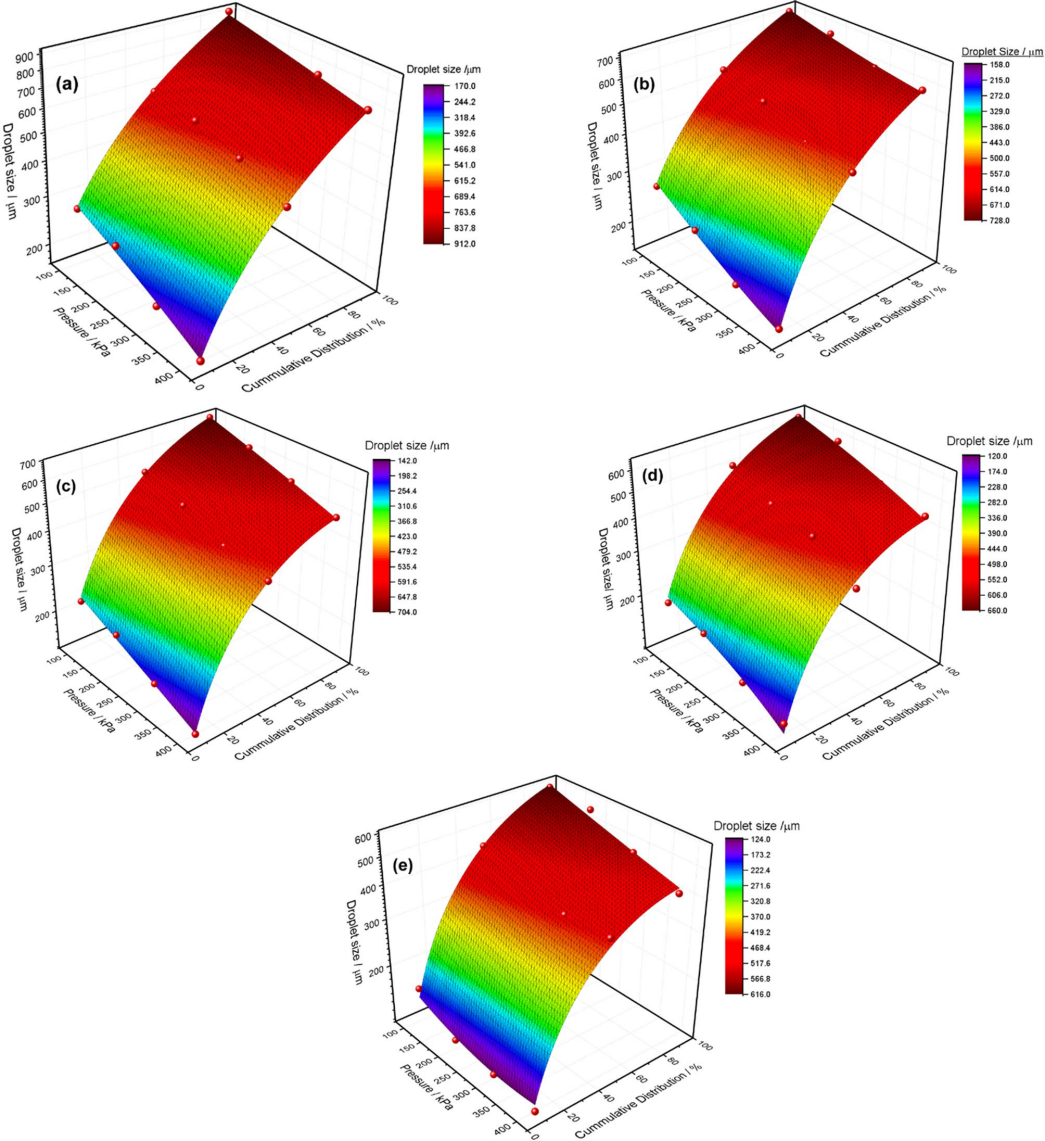
Effect of V-cut depth and pressure on droplet size and cumulative distribution. **(a)** Droplet size of A2-Model. **(b)** Droplet size of B2-Model. **(c)** Droplet size of C2-Model. **(d)** Droplet size of D2-Model. **(e)** Droplet size of E2-Model.

The size of pesticide droplets is an important factor affecting the effectiveness, safety, and environmental impact of pesticide use. The droplets classify fine aerosol droplets into two categories: fine and ultra-fine. It provides adequate coverage of plant surfaces but is less effective in inaccessible areas, such as under leaves, and it can penetrate dense plants more effectively. However, they are also vulnerable to wind-induced drift, increasing the risk of contamination in non-target areas, such as nearby crops or aquatic environments. The larger droplets lower the potential for drift, which leads to reduced harm to the environment and is more effective at covering small, open areas; plant surfaces may attempt to conceal or conceal evenly. Under hot and windy conditions, small droplets will evaporate quickly, reducing the contact time of the pesticide with the target. Nonetheless, larger droplets hold moisture longer, improving its effectiveness. Ultimately, the medium droplet size depends on the specific application, target pests, and environmental conditions, balancing effective coverage with minimal drift and environmental impact. In this study, it was observed how the depth of the V-Cut and pressure affect the spray droplet size. The study demonstrated that increasing the depth of the cut consistently reduces the angle of the nozzle’s V-Cut. This results in an increase in the nozzle’s convergence on the V-Cut. In this study, five models, ranging from A2 to E2, were experimentally investigated for their droplet sizes. Each model has a different depth of cut, which also affects the angle, as shown in Table 1. The produced spray droplet sizes were investigated at three different percentages: DV0.1, DV0.5, and DV0.9. DV0.1 represents the 10% of the total volume of the spray, DV0.5 represents the 50% of the total spray volume (also referred to as the median), and DV0.9 represents the 90% of the total spray volume. Experimental investigation of droplet size revealed that the A2 depth-of-cut nozzle produces a coarse droplet distribution. The median volume droplet diameter (Dv0.5) for A2 was observed to range between 600 microns and 400 microns, across pressure levels from 100 kPa to 400 kPa, respectively as shown in Fig. 5a. Additionally, the Dv0.9 values were recorded in the range of 900 microns to 700 microns. This behavior can be attributed to the A2 nozzle’s depth-of-cut of 0.75 mm, coupled with a V-cut angle of 76° as shown in Table-1, which results in reduced shear forces between the fluid stream and the nozzle surface. The decreased shear force reduces the breakup intensity of the liquid stream, thereby increasing the propensity for larger, coarse droplets. In this study, it was observed that as the depth of the V-Cut was increased, the droplet size consistently decreased, as demonstrated in Fig. 5b, c and d, and 5e. This relationship indicates a direct impact of the V-Cut geometry on droplet production and disintegration. The study emphasized that both the depth of the V-Cut and the applied pressure are critical factors in determining droplet size. The effect of pressure is especially important because it modifies the shear force applied to the fluid stream. This affects the atomization process further. Several studies stated that more significant than 200 μm is an adequate droplet size for several reasons. Such as D. Nuyttens et al. stated that for droplets smaller than 200 μm, there was no effect of variations in operating pressure on the droplet velocities, mainly because small droplets have small stopping distances and lose their initial velocity relatively quickly. It also stated that droplet size is more significant than 200 μm in diameter, where an increase in operating pressure increases velocities. All this information is very useful with regard to crop canopy penetration, the risk of spray drift, and the quantity and distribution of the deposit on the target^27^. Similarly, another study also reveals that medium droplet sizes (200 μm to 300 μm) are suitable for better coverage and versatility in reducing drift, especially in applications with varying pressure settings^28^. This range of sizes is considered appropriate for providing efficient spray performance and the desired engagement with the environment. In this study, the average droplet size of E2 was estimated to be within the range of 450 μm to 360 μm when the pressure was set at 100 to 400 kPa respectively. This change in droplet size with pressure is because when the pressure is increased this will increase the energy that is transferred to the fluid flow. This makes the breakup mechanism more intensified. One of the most important factors affecting this process is the V-Cut Angle, which is set to 36°. The angle of the V-Cut is important because it affects the flow dynamics at the point of contact between the fluid flow and the surface. However, at this particular angle the develop shear stress higher occurs between the fluid flow and the V-Cut surface, which plays an important role in droplet fragmentation. Shear stress at the interface causes turbulence and creates a local pressure gradient. This leads to further fragmentation of the spray droplets. This mechanism highlights the importance of optimizing both the V-Cut depth and pressure to achieve a finer and more controlled droplet size, which is essential for applications that require precise atomization, such as in pesticides spraying, fuel injection systems and spray coating processes. Very limited literature exists that discusses the development of a mathematical model capable of predicting droplet size. In this study, experimental results obtained from all developed nozzles were used to develop a mathematical model. Generally, a 2D nonlinear method is applied in developed models using a single independent variable. However, since this study found a close relationship between droplet size, depth of cut, and pressure, a 3D nonlinear surface fitting method was employed to develop the mathematical model. The non-linear poly2d function has been used to develop a mathematical model using the surface fitting method. The mathematical model that has been formulated in this study for estimating the droplet size requires three such variables, as stated in Eq. (12) and listed in Table 2. It is crucial to know that these three variables include h, which is the depth of the V-Cut, ∆P, which is the pressure, and DV%, which is the percentage of spray volume. The model was fitted well with an R^2^ of 0.96 indicating that the model can well estimate droplet size from the given parameters. The model is therefore effective in explaining and controlling the various characteristics of aerosols through the incorporation of multiple variables.

12 $$\begin{aligned} Droplet~size & =~z0+a \times h \times \Delta P+b \times D{v_\% } \\ & \;\;\;+c \times {\left( {h \times \Delta P} \right)^2}+d \times D{v_\% }^{2} \\ & \;\;\;+f \times h \times \Delta P \times D{v_\% } \\ \end{aligned}$$

**Table 2:** Droplet size prediction model fitted parameters.

Sr No.	Details	Parameters	Values	Standard error
1	Depth of V-Cut	h	From 0.75 to 1.75 mm	-
2	Change in pressure	$\Delta P$	From 100 to 400 kPa	-
3	Cumulative Distribution Value	$D{v_\% }$	10 to 90%	-
4	Constant	$z0$	198.09299	29.2202
5	Constant	*a*	-0.51027	0.14109
6	Constant	*b*	9.84897	0.7999
7	Constant	*c*	4.91864$\times {10^{ - 4}}$	1.76803$\times {10^{ - 4}}$
8	Constant	*d*	-0.02689	0.0072
9	Constant	*f*	-0.00503	9.78287$\times {10^{ - 4}}$

### Impact of V-cut depth and pressure on the quality of the spray coverage and volume distribution

**Fig. 6:**
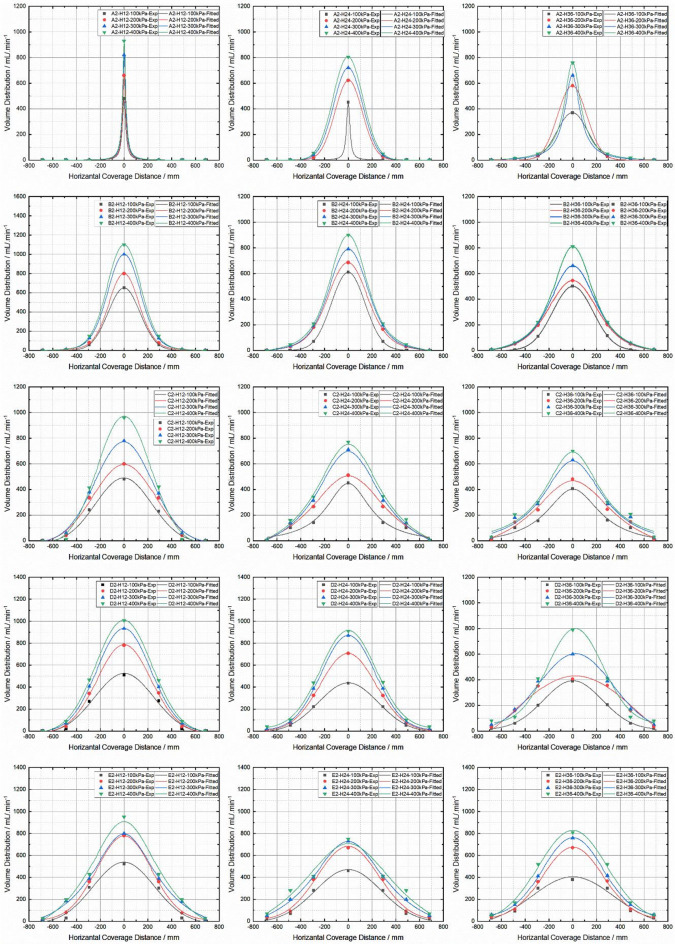
Spray volume distribution on difference V-Cut depth, Pressure and Height.

The importance of spray volume distribution in insecticide spraying is critical to ensure effective pest control while minimizing environmental impact. Proper distribution ensures even coverage whereas this allows the pesticide to reach all parts of the target surface such as hard-to-reach areas (under leaves) where insects often hide. In addition, a well-distributed spray volume reduces drift and spray loss that is critical issue of environmental pollution. By controlling droplet size and spray volume, the pesticides are reached to confined to the target area, reducing wastage and preventing off-target impacts. This not only increases the efficiency of pesticide use. But also reduces environmental impact by reducing the potential for pesticide contamination of soil, water and non-target organisms. The Well-managed spray distribution is essential to pest control effectiveness and sustainability of agricultural practices. Experiments were conducted in this study to determine the spray volume distribution at different heights and pressures. The spray nozzle is placed exactly over the bowls used for volumetric collection. The total horizontal distribution distance is 1,366 mm, equally distributed between the left and right nozzle positions as seen in Fig. 3. The volume distribution for five nozzles, from A2 to E2, was investigated at three different heights: As shown in Fig. 6, H12, H24, and H36. A fitting method was used to analyze the distribution of the spray volume to understand how the horizontal spray volume distribution occurs. Each nozzle and run for one minute, and the volume of spray collected in the bowl was measured. The results of this approach were a detailed analysis of the spray pattern and the effects of height and pressure on volume distribution. In this study, it was found that the spray volume distribution of nozzle A2 was very concentrated at a height of H12. The nozzle’s water jet like discharge pattern produced a very narrow spray angle, which reduced the spray breakup efficiency, leading to this behavior. The smaller spray angle decreased the fluid’s capability to atomize, and the majority of the droplets produced were captured in the bowl under the nozzle. This V-Cut Depth is likely to be less efficient for breakup due to the lower shear forces and higher nozzle velocity, resulting in coarse and more concentrated spray at close range are produced. A noticeable shift in the horizontal distribution of the spray volume was observed as the height between the nozzle and the collection bowl was increased. The reason for this change is that the distance between the nozzle and the collection target is increased. The spray spread more, reduces the concentration of the droplets around the nozzle, and spreads over a wider area with less concentration.

Furthermore, the study revealed that nozzles A2 and B2 displayed maximum spray volume at the center of the distribution pattern, with significantly lower spray volumes at the edges. For example, for B2 at H24, the spray volume at the center ranged between 600 and 900 ml/min at pressures of 100 kPa to 300 kPa, respectively. In this study also reveals that under the same pressure conditions, the spray volume decreased to a range of 200 to 60 ml/min at a distance of 300 mm from the center. That shows a normal spray pattern; the bulk of the volume is concentrated at the center of the nozzle and further away from the nozzle, the number of droplets and density of the droplet decreasing accordingly because of gravitational forces and aerodynamic forces, which makes the spray spread out and lose momentum.

Additionally, the D2 nozzles at H24 and H26, as well as the E2 nozzles at H12, H24 and H36, have a more uniform and efficient spray distribution. It shows the significance of nozzle that the volume distribution is consistent and gradual decreasing from center of the nozzle to the end. This clearly shows that the designs or positions of these nozzles and targeted areas are not only intended to spread the pesticide evenly over a larger area but also to produce the best spray distribution. If spray volume decreases gradually, it means good penetration and distribution, adequate coverage and a more efficient application of pesticides.

In Fig. 6, the results of D2 at H24 demonstrate that at 300 mm away from the center, the spray volume collected was approximately half of the volume collected at the center. This gradual decline in volume with increasing distance from the nozzle indicates a well-optimized spray pattern where the droplet size remains relatively consistent across the area, but the density of droplets decreases gradually, ensuring better overall coverage and reducing the likelihood of over-application or pesticide waste. This is a key feature of a well-calibrated nozzle, where the spray characteristics, including droplet size, distribution, and volume, are precisely controlled to maximize pesticide effectiveness while reducing environmental impact.

### Impact of V-Cut depth and applied pressure on spray angle and sheet length formation

**Fig. 7:**
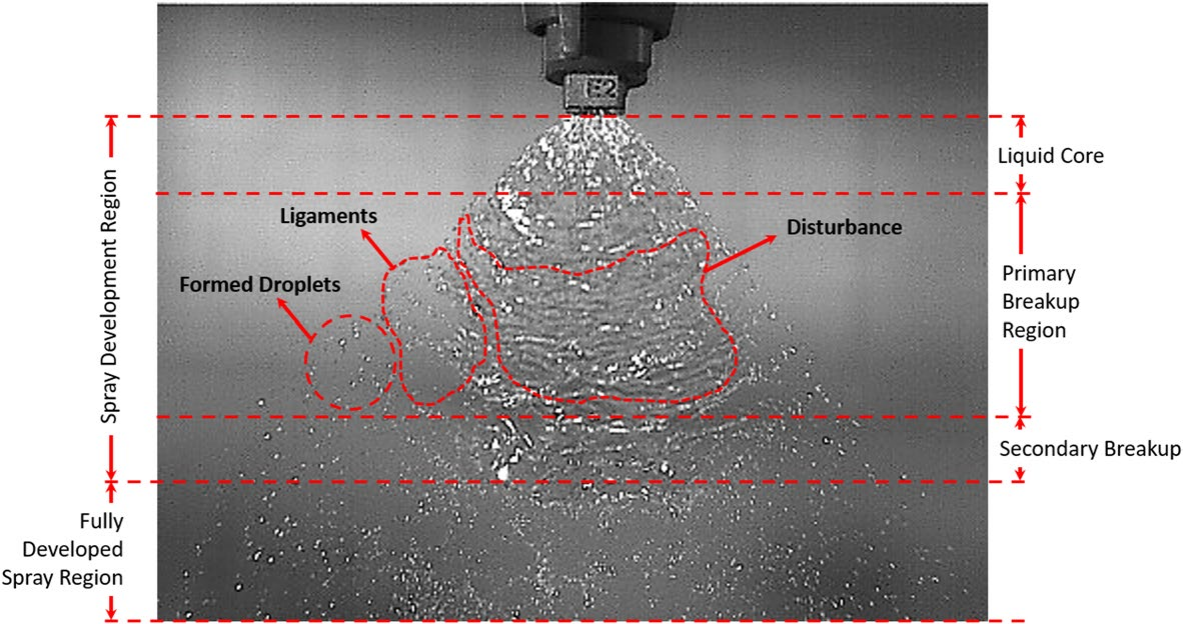
High speed captured image for identification the regions of produced spray from developed nozzle.

**Fig. 8:**
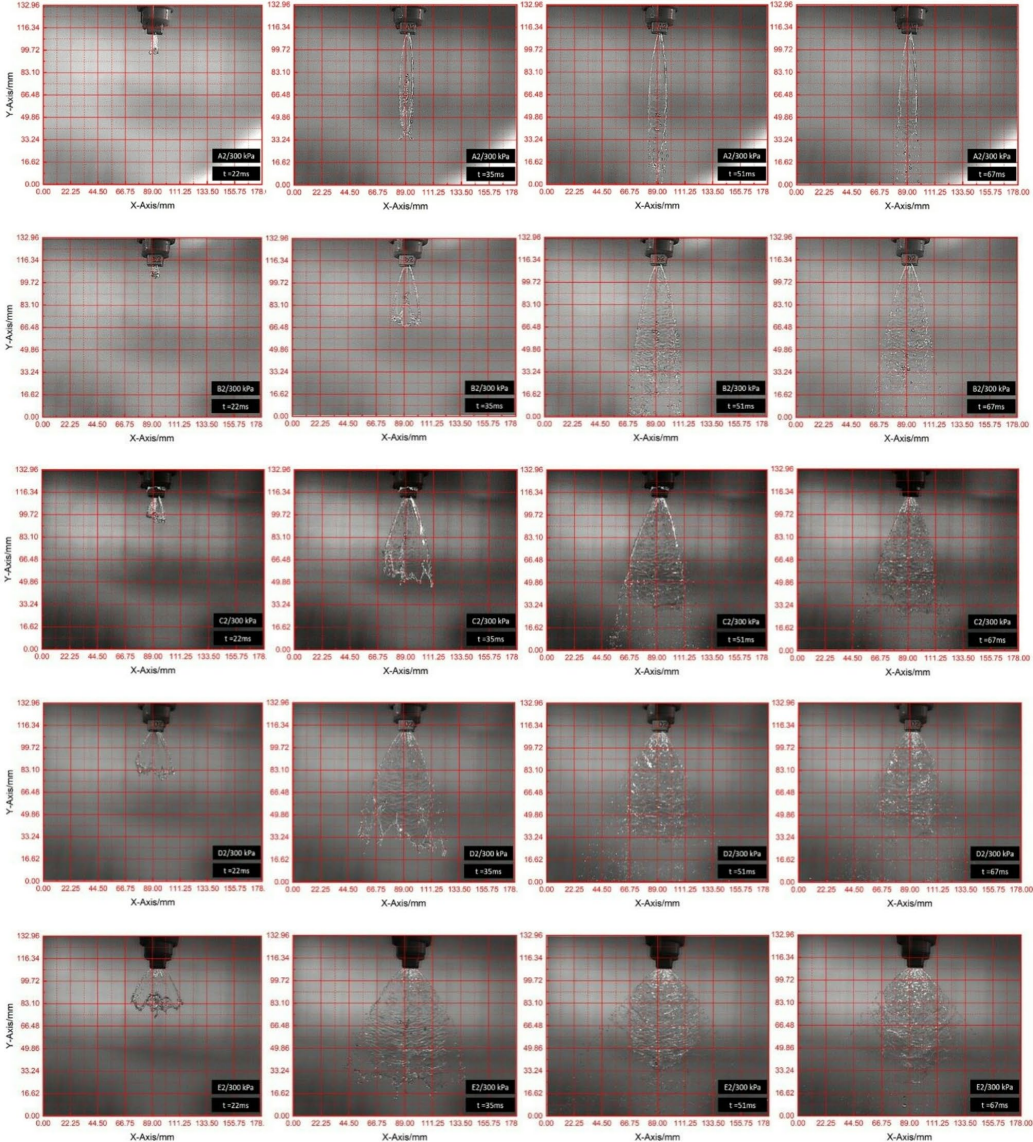
Images captured on different time frames to measure the spray angle and sheet length.

**Fig. 9:**
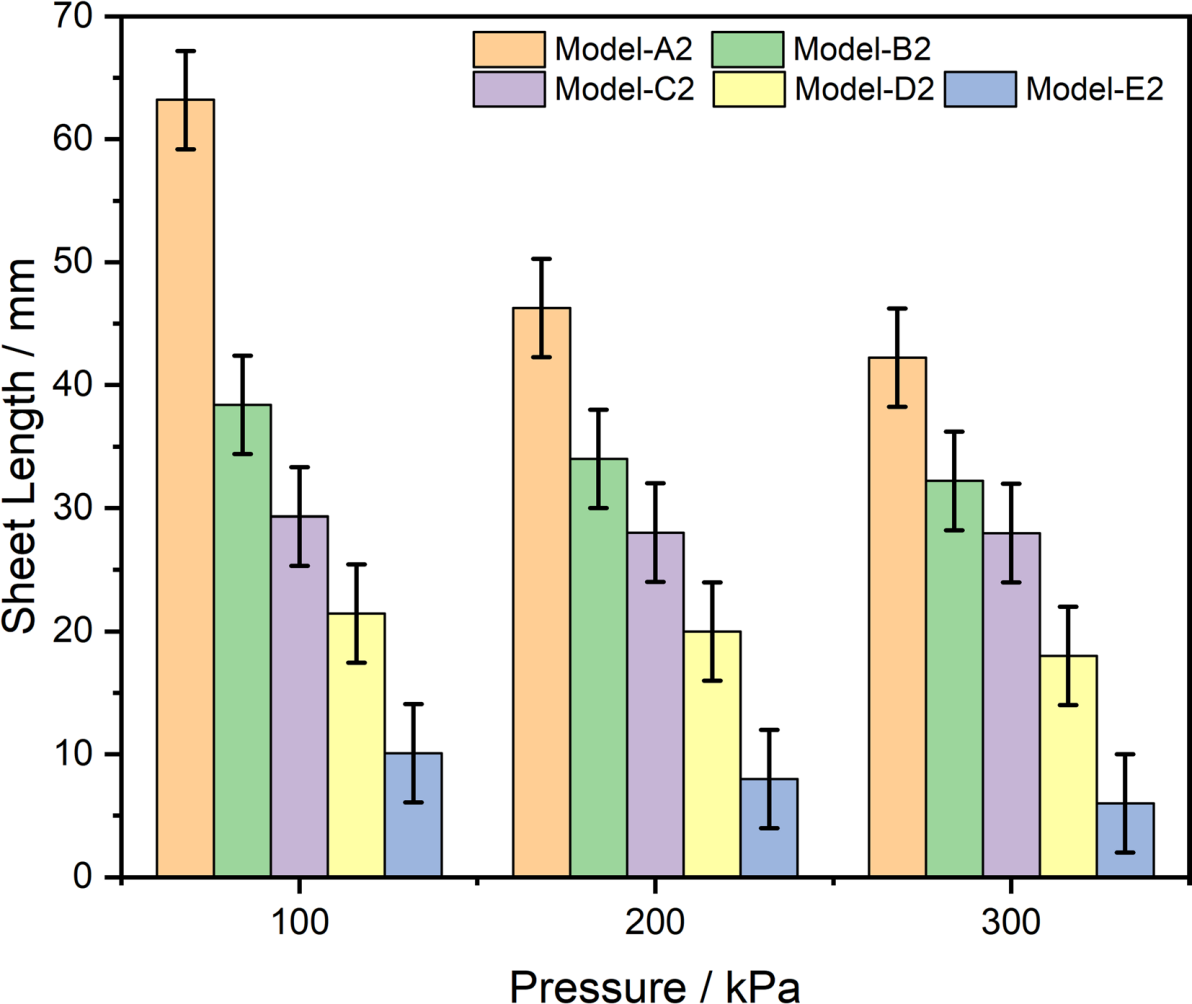
Impact of V-cut depth and pressure on Sheet Length.

The spray formation region is where the liquid jet experiences its primary and secondary breakup processes, resulting in the formation of a spray of droplets, as shown in Fig. 7. This region typically begins immediately after the liquid exits a nozzle or orifice and extends through the initial stages, during which the jet becomes unstable and starts to develop droplets. Aerodynamic forces including shear of surrounding air and of a liquid and the relative velocity of liquid and air are all important in the droplet breakup process in this region. These factors allow ligaments to separate into small droplets. The spray formation zone is characterized by different droplet sizes which is affected by various factors such as nozzle design properties of the fluid (viscosity, surface tension) and the air surrounding the fluid. The liquid’s end of this region is when the liquid has been fully atomized into small droplets and uniform to serve the intended application, for example fuel spray in internal combustion engine, agricultural spraying, or spray coating. The influence of V-cut depth and pressure on spray angle and sheet length was investigated in this study to understand better their impact on spray characteristics seen in Table 3; Fig. 9 respectively. To examine these parameters in detail, a high-speed camera was employed, as discussed in section “Spray angle and sheet length measurement methodology”. The spray characteristics from five different V-cut depths were analyzed at three different pressures—100 kPa, 200 kPa, and 300 kPa—to provide a comprehensive understanding of their effects. Results showed that the A2 model nozzle had a significantly lower spray angle, which can be attributed to the specific geometry of the V-cut. The V-cut angle in the A2 nozzle was 76°, resulting in a disturbance in the atomization process and improper droplet breakup. The resulting phenomenon is jet like behavior rather than the expected fan shaped spray pattern. The V shaped cutting angle is too shallow to disturb the liquid stream properly, resulting in the formation of a liquid jet and preventing proper atomization of spray. On the other hand, the spray angle increased progressively with the increase of the V-cut depth. This result is logical, as a improve V-Cut depth in nozzle permits more divergence of the fluid stream as it leaves the nozzle, scattering the liquid over a further area and increasing the spray angle. Figure 8; Table 2 demonstrate this correlation between increased V-cut depth and a broader spray angle. The study also showed a strong correlation between V-cut depth and sheet length. The experimental results showed that the sheet length was obtained maximum of 64 ± 4 mm with the A2 nozzle at 100 kPa pressure. The reason is that the fluid stream exits the nozzle with a lower velocity at lower pressure, and the fluid stream travels a long distance before forming droplets. However, as the pressure was increased, the spray sheet length decreased, with values ranging from 48 mm ± 4 to 45 ± 4 mm as pressure increased from 100 kPa to 300 kPa. There is a well-known phenomena that occurs when the sheet length decreases as the pressure increases. This is because an increase in pressure resulted higher velocity at the nozzle exit position. The high exit velocity leads to faster droplet breakup and hence reduced length of the spray sheet. Additionally, the increased velocity also decreases spray effective distance, allowing droplets to spread out more rapidly.

Furthermore, the study revealed that the shortest sheet length formation was observed with the E2 nozzle, with measurements of 10 ± 3 mm at 100 kPa, 8 ± 3 mm at 200 kPa, and 7 ± 3 mm at 300 kPa as shown in Figs. 8 and 9. This implies that the nozzle probably created a more concentrated and high-pressure flow but one that was rapidly broken up into drops. Rapid breakup created a smaller the primary breakup region, which formed spray droplets rapidly but nearer to the nozzle. This behavior suggests that the E2 nozzle produced a higher shear force between the fluid stream and the nozzle wall, which intensified with increasing V-cut depth. It was found that the breakup of the liquid into fine droplets was due to the shear stress in the nozzle. The nozzle’s ability to induce higher shear stress, resulting in faster breakup of the liquid stream and shorter sheet lengths, increases as the V-cut depth increases. Finally, this study offers a detailed technical understanding of the interaction between V-cut depth, pressure, and spray characteristics. The results are justified by considering the fluid dynamics of atomization, shear stress interactions, and the effect of nozzle geometry on spray behavior. The findings indicate how V-cut depth and pressure settings can affect desirable spray performance under various applications.

The mathematical model developed in this study is based on experimental data about the spray angle, which is used to predict this parameter accurately as seen in Eq. (13). The model employs two independent variables: pressure and V-Cut depth, which have been identified as critical factors influencing the spray angle. The V-Cut depth is varied between 0.75 and 1.75, and pressure is varied between 100 and 300 kPa in this model. The variables selected were those that directly affect the spray characteristics and are of practical importance in the system under investigation. Table 3 shows that the predicted values from the model are in high degree of consistency with the experimental data, which confirms the robustness of the model.

13 $$Spray~Angle~\left( \theta \right)=~ - 42.42+0.0085 \times \Delta P+72.067 \times h$$.

**Table 3:** Comparison of spray angle experimental results with predicted value produced from developed mathematical model.

Sr. No	Model	Variation in spray angle due to pressure								
		Spray angle on 100 kPa			Spray angle on 200 kPa			Spray angle on 300 kPa		
		Exp	Pred	Error %	Exp	Pred	Error %	Exp	Pred	Error %
1	A2	10^o^	12.48^o^	+ 19.8	12^o^	13.33^o^	+ 10.00	14^o^	14.18^o^	+ 1.29
2	B2	32^o^	30.50^o^	-4.91	33^o^	31.35^o^	-5.26	34^o^	32.20^o^	-5.58
3	C2	46^o^	48.51^o^	+ 5.18	47^o^	49.36^o^	+ 4.79	49^o^	50.21^o^	+ 2.42
4	D2	66^o^	66.53^o^	+ 0.80	67^o^	67.38^o^	+ 0.56	67^o^	68.23^o^	+ 1.80
5	E2	85^o^	84.55^o^	-0.53	87^o^	85.40^o^	-1.87	87^o^	86.23^o^	-0.86

### Influence of depth of V-cut on Weber number, velocity and in coefficient of discharge

**Fig. 10:**
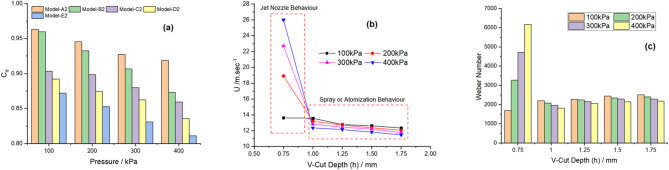
Impact of pressure and V-cut depth on coefficient of discharge, velocity and Weber number. **(a)** Coefficient of Discharge. **(b)** Velocity. **(c)** Weber Number.

The performance of flat fan nozzles depends significantly on the coefficient of discharge (Cd), velocity and Weber number (We), which are fundamental parameters in fluid dynamics and spray applications. The coefficient of discharge is the efficiency with which a nozzle converts pressure energy into kinetic energy. To achieve low energy losses and stable spray patterns, it is necessary to find out the actual flow rate through the nozzle and adapt the nozzle geometry to specific applications. Another parameter which has an effect on both atomization and droplet size is the velocity of the fluid leaving the nozzle. The We number is another dimensionless parameter, which can characterise the importance of the inertial forces compared with the surface tension effect for the breakup of the fluid stream. The use of the Weber number also shows that when the value is high, inertial forces take over, cause the formation of finer droplets and good atomization whereas when the value is low this leads to surface tension hence formation of larger droplets. These parameters are interlinked, as velocity can improve the Weber number and atomization, but Cd, which is a measure of nozzle efficiency, is critical in achieving the best spray performance. In combination with the coefficient of discharge, velocity, and Weber number define the features of the spray, as well as its efficiency and efficacy in practical contexts such as fuel combustion, agriculture with irrigation and pesticide application, and industrial washing. In this study, the impact of pressure on the V-Cut depth of five nozzles was examined in relation to the coefficient of discharge, velocity, and Weber number. The results indicated that nozzle A2 exhibited the highest coefficient of discharge at 100 kPa. However, as pressure increased, the coefficient of discharge progressively decreased. This phenomenon can be attributed to the reduction in V-Cut depth at higher pressures, which led to a reduction in frictional losses, thereby enhancing the actual discharge. Reduced frictional resistance leads to enhanced flow rates at lower pressures and an increase in the efficiency of discharge. On the other hand, as the depth of the V-Cut was increased, the angle of the V-Cut was more convergent and the flow was more turbulent and the frictional losses were higher. When frictional losses increased the flow was more restricted and less efficient hence reducing the discharge coefficient. The reduction in actual discharge, caused by the rise in frictional resistance, is evident in Fig. 10a. This shows that frictional losses are inversely proportional to the discharge efficiency, and how the geometry of the nozzle, particularly the V-Cut depth, affects flow characteristics and performance under different pressure. This research provides a thorough analysis of the velocity profiles of five different nozzles. The A2 nozzle had the highest velocity as shown in Fig. 10b. This was due to the shallow V-cut depth which was similar to the effect as water jet nozzle and hence a more concentrated flow. Consequently, the flow was more directed, which resulted in a smaller spray angle. A small increase in the depth of the cut drastically reduced the velocity. This reduction occurs due to a corresponding increase in the outlet area. The deeper cut implies a larger discharge area, which in turn results in a lower velocity, as the same volumetric flow rate must traverse a greater area. In this study, the Weber number was calculated to compare the inertial forces with the surface tension in the field of fluid dynamics. The Weber number is also a dimension less number which gives the ratio of inertial forces to surface tension in a fluid flow. The results indicate that the Weber number for the A2 model is significantly high, which can be attributed to the relatively shallow depth of cut. A smaller depth of cut means that inertial force is relative to surface tension of water hence the higher Weber number is attributed. Furthermore, the study demonstrates that an increase in fluid stream velocity corresponds to a simultaneous rise in the Weber number, as evident in Fig. 10c for the A2 model. This is in line with the theoretical experience that, when velocities are higher, inertial forces experience by the fluid are higher and proportionally are more significant compared to surface tension forces. Additionally, the study shows that increasing the depth of the V-cut leads to a slight increase in the Weber number, suggesting a direct influence of cut geometry on the balance between inertial and surface tension forces. An increase in pressure reduces the Weber number because the fluid becomes more convergent, diminishing the effect of inertial forces. Among the models B2 to E2, the highest Weber number is observed in the E2 model, primarily due to the enhanced spray production. On the other hand, Reynolds number has direct relation with pressure so higher value of pressure result in lower Weber number. This may be because the fluid is more converge thus reducing the effect of inertial force which lead in reduction.

## Conclusion

Droplet size impacts nozzle efficiency, waste reduction, and environmental impact. V-cut depth considerably impacts flat fan nozzle spray pattern and droplet size distribution. A “V”-cut nozzle design beveles orifice edges to produce a fan-like spray pattern. This study found that models C2, D2, and E2 covered 100, 200, and 300 kPa of spray volume well. In this study, Five V-shaped cutting depths of a flat fan nozzle were evaluated to see how they affected spray properties such droplet size, spray volume distribution, spray angle, speed, length, discharge coefficient, and Weber number and velocity. As the depth of the V-cut decreased as in A2, the angle became 76°, decreasing the spray angle. This short depth of cut created a smaller outlet area, which gave the nozzle jet-like behavior and increased velocity. This reduced shear force, resulting in coarse droplets. However, as the V-cut depth increased, the spray angle improved. This improvement occurred because the V-cut angle converged with increased V-cut depth, which enhanced shear stress. The improvement in shear stress led to finer droplets. The study also showed that the volume distribution improved with increased V-cut depth, which can lead to better pesticide coverage. The coefficient of discharge for A2 was the most investigated, with the primary reason being the reduction in the difference between actual and theoretical discharge. Additionally, two mathematical models were developed: one to predict the produced droplet size and the other to predict the spray angle. The droplet size model used three independent variables—V-cut depth, pressure, and DV percentage—while the spray angle model used pressure and V-cut depth as independent variables. Both developed models showed excellent agreement with the experimental results. This study suggests that the V-cut depths of Model-C2, D2, and E2 are ideal because they result in a good spray angle, excellent coverage, and droplet size. As in this work, the V-cut depth can impact the spray properties; likewise, the opening gap of the nozzle can also affect the spray characteristics. This feature should be investigated more in the next research.

## Data Availability

The datasets used and/or analyzed during the current study are available from the corresponding author on reasonable request.
